# Association between Psychosocial Factors and Oral Symptoms among Residents in Fukushima after the Great East Japan Earthquake: A Cross-Sectional Study from the Fukushima Health Management Survey

**DOI:** 10.3390/ijerph18116054

**Published:** 2021-06-04

**Authors:** Narumi Funakubo, Ayaka Tsuboi, Eri Eguchi, Fumikazu Hayashi, Masaharu Maeda, Hirooki Yabe, Seiji Yasumura, Kenji Kamiya, Shogo Takashiba, Tetsuya Ohira, Mental Health Group of the Fukushima Health Management Survey

**Affiliations:** 1 Department of Epidemiology, Fukushima Medical University School of Medicine, Fukushima 960-1295, Japan; naru-23@fmu.ac.jp (N.F.); fhayashi@fmu.ac.jp (F.H.); teoohira@fmu.ac.jp (T.O.); 2 Department of Pathophysiology-Periodontal Science, Okayama University Graduate School of Medicine, Dentistry and Pharmaceutical Sciences, Okayama 700-8558, Japan; aya.t1219@s.okayama-u.ac.jp (A.T.); stakashi@okayama-u.ac.jp (S.T.); 3 Radiation Medical Science Center for the Fukushima Health Management Survey, Fukushima Medical University, Fukushima 960-1295, Japan; masagen@fmu.ac.jp (M.M.); hyabe@fmu.ac.jp (H.Y.); yasumura@fmu.ac.jp (S.Y.); kkamiya@fmu.ac.jp (K.K.); 4 Department of Disaster Psychiatry, Fukushima Medical University School of Medicine, Fukushima 960-1295, Japan; 5 Department of Neuropsychiatry, Fukushima Medical University School of Medicine, Fukushima 960-1295, Japan; 6 Department of Public Health, School of Medicine, Fukushima Medical University, Fukushima 960-1295, Japan; 7 Research Institute for Radiation Biology and Medicine, Hiroshima University, Hiroshima 734-8553, Japan

**Keywords:** great East Japan earthquake, disaster, oral symptoms, psychosocial factors, evacuation, cross-sectional study

## Abstract

Oral health is closely related to subjective general health and systemic diseases. This cross-sectional study aimed to identify the factors related to oral symptoms and their worsening in relation to psychosocial factors after the Great East Japan Earthquake. In this study, 64,186 residents aged 15–101 years old, who experienced the earthquake on 11 March 2011, were surveyed regarding their oral symptoms; psychological factors, such as post-traumatic reactions and psychological distress; and social factors such as evacuation, work change, and loss of a close person; history of systemic diseases; and lifestyle. Binomial logistic regression analysis was used to calculate odds ratios, and 95% confidence intervals were established for each factor associated with prevalent and exacerbated oral symptoms. The proportions of participants with prevalent and exacerbated oral symptoms were 10.3% and 1.6%, respectively. The multivariate odds ratios and 95% CI of psychosocial factors associated with exacerbated oral symptoms were as follows: post-traumatic stress disorder symptoms, 2.24 (1.64–3.06); work changes, 1.88 (1.34–2.65); history of dyslipidemia, 1.74 (1.27–2.39); and subjective current poor health condition, 2.73 (2.00–3.75). Psychological factors, social factors, and physical factors were associated with both prevalent and exacerbated oral symptoms.

## 1. Introduction

Disasters have a great impact on the lives and health of evacuees. Residents of Fukushima Prefecture were forced to evacuate for an extended time following the Great East Japan Earthquake on 11 March 2011, and the subsequent tsunami and nuclear accident at the Fukushima Daiichi Nuclear Power Plant. These disasters have hindered the evacuees not only at the time of the disaster but also afterward by affecting both their physical [1,2] and mental conditions [3,4,5]. Six to eleven months after the Great East Japan Earthquake, 42.6% of the evacuees had moderate or severe mental health problems [6]. Furthermore, the mental health problems and trauma reactions of the evacuees continued for several years after the disaster [7], and problems were manifested in the body, particularly for the evacuees and unemployed people [8].

The oral cavity is involved in eating and communication. Oral health has been associated with subjective general health [9,10], and periodontal disease has been associated with systemic diseases such as diabetes, dyslipidemia, stroke, heart disease, and pneumonia [11]. Maintenance of good oral health is important because it prevents oral symptoms from worsening, improves subjective general health, and might prevent dyslipidemia and diabetes from worsening. The direct oral health problems observed immediately after a disaster include deterioration of oral hygiene caused by poor cleaning related to lack of water, as well as deterioration of oral functions due to loss of dentures [12]. Subsequent indirect problems include dental caries and periodontal disease related to unbalanced diets and the stress of prolonged evacuation. In a previous study in Otsuchi-cho, Iwate, Japan, Kishi et al. reported that during the Great East Japan Earthquake [9], age, loss or damage of dentures, having fewer than 20 teeth, untreated teeth, and tooth mobility were factors associated with lower oral-health-related quality of life (OHRQOL). In addition, oral symptoms, including toothaches, have been found to be related not only to actual caries and periodontal disease but also to various other factors such as psychological problems, environment, and lifestyle [13,14]. People who lived in temporary housing after the Great East Japan Earthquake had a significantly higher prevalence of toothache [15].

Given the mental distress and decline in oral hygiene following large-scale disasters, as well as the association of subjective general health with oral health, it is very important to understand the relationship between psychosocial factors and oral symptoms following disasters such as the Great East Japan Earthquake. To date, however, there have been few large-scale studies that have focused on this relationship. The purpose of this study was to identify factors related to oral symptoms, such as toothache and gingival inflammation, and their worsening in terms of mental health factors such as disaster-related psychosocial and lifestyle factors, and the general medical history of residents of the evacuation zone in Fukushima Prefecture after the Great East Japan Earthquake. The findings of this study are likely to be useful for oral and mental health considerations following future disasters.

## 2. Materials and Methods

### 2.1. Participants

**Figure 1:**
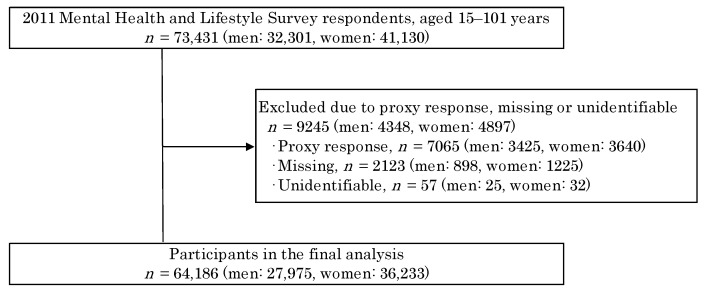
Flowchart of study participants after the Great East Japan Earthquake.

In this cross-sectional study, the Mental Health and Lifestyle Survey was conducted as part of the Fukushima Prefecture Health Care Survey among 64,186 participants who were born on or before 1 April 1995, i.e., those who were approximately 16 years old or older at the time of the Great East Japan Earthquake. The details of this survey are described in previous studies [2,16]. Briefly, the survey was conducted by sending self-administered questionnaires to approximately 210,000 people who were registered as residents in evacuation areas at the time of the earthquake and divided into five age groups (0–3 years old, 4–6 years old, elementary school students, junior high school students, and people approximately 16 years old or older) according to their current mental and physical health, lifestyle, and living conditions. The survey was conducted from 18 January 2012, to 31 October 2012, approximately 10 months after the earthquake occurred on 11 March 2011. A total of 73,431 out of 180,604 people aged approximately 16 years and above responded (response rate, 40.7%) [17], of which proxy responses (*n* = 9245) were excluded. Of the respondents, 64,186 evacuees aged 15–101 years (mean age: 55.2 years) were included in the analysis (Figure 1).

This study was conducted in accordance with the provisions of the Declaration of Helsinki. The study protocol was approved by the Fukushima Medical University Ethics Committee (approval number 1316), and a questionnaire was mailed to the participants stating the purpose of the study and that it would be used for analysis. By returning the questionnaire, participants were considered to have given their written consent to participate.

### 2.2. Measurements

#### 2.2.1. Oral Symptoms

All the participants were asked to answer the multiple-choice question (Q5 in Supplementary Material S1), “Have you felt unwell in the past several days due to illness or injury etc.?” If they answered yes, then we asked, “What kind of symptoms have you had? Please circle all applicable symptoms and draw a double circle around any symptoms that have worsened since the disaster.” Thus, answer choices were “none,” “yes,” and “yes + worsened since the disaster” for the symptoms of toothache or swollen/bleeding gums. If the participant circled for any of the oral symptoms, we assessed that the participant had oral symptoms. If the participant drew a double circle “worsened since the disaster” to any of the questions on oral symptoms, we assessed that the participant had worsened oral symptoms. Thus, the participants were classified into three groups: without oral symptoms (absent), with oral symptoms (prevalent), and worsened oral symptoms (exacerbated). The questionnaire for this study is available in Supplementary Material S1.

#### 2.2.2. Psychological Factors

Psychological factors that may influence oral symptoms were evaluated as psychological distress (K6: Kessler Psychological Distress Scale) [18,19] and post-traumatic stress disorder (PTSD: PCL-S; PTSD Check List Stressor-Specific Version) [20,21].

The K6 scale was used to assess psychological distress [18]. This scale asks participants whether they have experienced any of the following six feelings during the past 30 days: “nervousness,” “hopeless,” “restless or inability to relax,” “inescapable sadness,” “worthless,” and “everything requires painstaking effort.” Each question was scored on a five-point Likert-type scale from 0 to 4. The scores ranged from 0 to 24, and higher scores indicated worse psychological distress. The Japanese K6 version has been validated and was used in this study [19]. Psychological distress was defined as K6 scores ≥13.

The PCL-S [20] was used to assess PTSD caused by the experience of the Great East Japan Earthquake and the following accidents. The PCL-S is a 17-item self-report measure designed to detect PTSD, where each item is scored from 1 to 5, corresponding to “not at all,” “slightly,” “moderately,” “quite a lot,” or “very much,” respectively. The scores ranged from 17 to 85, and higher scores indicated worse PTSD symptoms The Japanese PCL-S version has been validated and was used in this study [21]. The presence of PTSD symptoms was defined as a PCL-S score ≥44.

#### 2.2.3. Experience of the Great East Japan Earthquake

Information was also collected on potential confounders such as the experiences of evacuation, earthquake, tsunami, or hearing the sound of explosions from the nuclear power plant accident, job loss, work changes since the disaster, and loss of a close person (no/yes), and house damage (major/minor or none). Experienced evacuees were defined those whose addresses at the time of the disaster were in nine towns or villages (Hirono Town, Naraha Town, Tomioka Town, Kawauchi Village, Okuma Town, Futaba Town, Namie Town, Katsurao Village, and Iitate Village) or residents of four cities or town (Tamura City, Minamisoma City, Kawamata Town, and Date City) who lived in shelters or temporary housing after the earthquake. The question on house damage was scored on a five-point scale from 1 to 5, defined as follows: 1 = no damage, 2 = partial damage, 3 = partial collapse, 4 = partial but extensive collapse, and 5 = total collapse. If the participant answered 4 or 5, we assessed the damage as “major”; otherwise as “minor or none.”

#### 2.2.4. Medical History and Current Health Condition

Information on history of mental illness, hypertension, diabetes mellitus, dyslipidemia (no/yes), and current health condition (good/normal/bad) was collected. Each participant’s current health condition was assessed as good if they answered “good” or “normal” and as poor otherwise.

#### 2.2.5. Lifestyle Factors and Other Confounding Factors

Results were collected on the following lifestyle factors: current smoking habit (no/yes), current drinking habit (no/yes), regular exercise habit (once a week or less/twice a week or more). Information on potential confounders, such as sex (men/women) and age category (49 years or younger/50–69 years/70 years or older), was also collected.

### 2.3. Statistical Analysis

The mean ages for the sex and age category and the proportions of nominal scale data were calculated. Two outcomes were considered: (i) prevalent oral symptoms (yes/no) was defined as endorsement of at least one oral symptom and did not include any oral symptoms also endorsed as “worsened since the disaster”; and (ii) exacerbated oral symptoms (yes/no) was defined as endorsement of at least one oral symptom as “worsened since the disaster.” Logistic regression models of each outcome were used to calculate odds ratios and their 95% confidence intervals (CIs) for the following factors: sex; age category; PTSD symptoms; experiences of evacuation, tsunami, hearing nuclear explosions, house damage, job loss, work change, and loss of a close person; histories of mental illness, dyslipidemia, hypertension, and diabetes mellitus; current health condition; smoking and exercise habits. Two models were used. Model 1 was run for each factor individually, while controlling for sex and age category. Model 2 had sex and age category as factors and all factors found to be statistically significant in Model 1; those factors not reaching statistical significance in Model 1 were not included in Model 2. The results of the multivariate adjustment analysis were presented using Model 2. As multicollinearity was found between the PTSD symptoms (from the PCL-S) and psychological distress (from the K6 scale), and the presence of PTSD symptoms was associated more with oral symptoms than with psychological distress, we presented the results of the multivariate adjustment analysis using the PTSD symptoms alone. We conducted stratified analysis by sex and age category (age 49 years or younger, age 50–69 years, age 70 years or older) with reference to previous literature [9]. SAS statistical software version 9.4 (SAS Institute Inc., Cary, NC, USA) was used for analyzing the data. *P*-values of less than 0.05 were considered significant.

## 3. Results

### 3.1. Characteristics of Participants and Prevalence of Oral Symptoms

The mean age of the participants was 55.2 years. The proportions of the participants with the measured factors were as follows: PTSD symptoms, 21.5%; psychological distress, 14.6%; experiences of disaster (evacuation 46.8%, tsunami, 20.3%; hearing nuclear explosions, 52.6%; house damage—half or more destroyed, 15.9%; job loss, 20.9%; work change, 55.7%; loss of a close person, 20.0%); history of systemic diseases (mental illness, 5.0%; hypertension, 41.2%; diabetes mellitus, 19.5%; and dyslipidemia, 35.8%); current poor health condition, 18.5%; and current lifestyle (smoking habit, 21.1%; drinking habit, 46.1%; and exercise habit—once a week or less, 64.7%). The characteristics of the participants and their oral symptoms according to the sex and age category are shown in Table 1. The proportion of participants with oral symptoms (prevalent) was 10.3% (by sex: men, 9.9% and women, 10.6%; by age category: 49 years or younger, 8.8%; 50–69 years, 11.4%; and 70 years or older, 10.6%) and was slightly higher for women and those aged 50–69 years. The proportion of participants with exacerbated oral symptoms was 1.6% (by sex: men, 1.4% and women, 1.8%; by age category: 49 years or younger, 1.8%; 50–69 years, 1.7%; and 70 years or older, 1.1%) and tended to be higher among those aged 69 years or younger.

### 3.2. Multivariate Adjustment Analysis of Psychosocial Factors

For prevalent and for exacerbated oral symptoms, Table 2 shows the odds ratios and 95% CIs for factors considered in Model 1 and in Model 2. Each group of prevalent and exacerbated oral symptoms was multivariable-adjusted (Model 1 and Model 2) using a logistic regression analysis, and each reference had no oral symptoms (absent).

#### 3.2.1. Prevalent Oral Symptoms

After adjustment for sex and age category (Model 1), the following factors were significantly associated with prevalent oral symptoms: psychological factor of PTSD symptoms; experience of disaster, including work change, hearing nuclear explosions, loss of a close person, job loss, evacuation, tsunami, and house damage; histories of systemic diseases, including mental illness, dyslipidemia, diabetes mellitus, and hypertension; current poor health condition; current lifestyle, including exercise habit (once a week or less) and smoking habit; and being female. After the multivariate adjustment (Model 2), PTSD symptoms; experiences of disaster, including work change, hearing nuclear explosions, and loss of a close person; histories of mental illness, dyslipidemia, and hypertension; and current poor health condition were significantly associated with prevalent oral symptoms.

#### 3.2.2. Exacerbated Oral Symptoms

After adjustment for sex and age category (Model 1), the following factors were significantly associated with exacerbated oral symptoms: psychological factor and PTSD symptoms; experiences of disaster, including work change, loss of a close person, job loss, evacuation, hearing nuclear explosions, house damage, and tsunami; histories of systemic diseases, including mental illness, dyslipidemia, and hypertension; current poor health condition; current lifestyle, including smoking and exercise habits (once a week or less); and being female. After the multivariate adjustment (Model 2), PTSD symptoms, work change, history of dyslipidemia, current poor health condition, and age categories (49 years and younger and 50–69 years) were significantly associated with exacerbated oral symptoms.

### 3.3. Multivariate Analysis for Prevalent and for Exacerbated Oral Symptoms on Psychosocial Factors by Sex and Age Category

For prevalent and for exacerbated oral symptoms, Table 3 shows the multivariate (Model 2) odds ratios and 95% CIs of the prevalent and exacerbated oral symptoms in the participants who fit for each factor as compared with those who did not fit each factor, obtained using a logistic regression analysis according to sex and age category. As a sensitivity analysis, the same analysis was performed by replacing PTSD symptoms with K6, and almost similar results were obtained.

#### 3.3.1. Prevalent Oral Symptoms for Each of the Sexes

In both men and women, significant association with the prevalent oral symptoms was found as follows: having PTSD symptoms, work change, histories of mental illness, dyslipidemia, and current poor health condition. There were significant associations between the experience of evacuation among men and the loss of a close person among women. No statistical evidence showed that tsunami experience, house damage, histories of hypertension and diabetes mellitus, and current smoking habit were related to the prevalent oral symptoms in both men and women.

#### 3.3.2. Exacerbated Oral Symptoms for Each of the Sexes

Similarly, significant associations with worsened oral symptoms (exacerbated) were found in both men and women as follows: having PTSD symptoms, work change, history of dyslipidemia, and current poor health condition. No statistical evidence supported that experiences of evacuation, tsunami, job loss, and house damage; histories of mental illness, hypertension, and diabetes mellitus; and current exercise habit were related to the exacerbated oral symptoms for both men and women.

#### 3.3.3. Prevalent Oral Symptoms for Each of the Age Category

In the age categories, significant associations with the presence (prevalent) of oral symptoms were found among all the age categories as follows: having PTSD symptoms, history of dyslipidemia, and current poor health condition. For ages 50 years and older, history of mental illness was associated with the prevalent oral symptoms. For ages 69 years and younger, work change was associated with the prevalent oral symptoms.

#### 3.3.4. Exacerbated Oral Symptoms for Each of the Age Category

In the age categories, significant associations were found between exacerbated oral symptoms for all age categories and PTSD symptoms and current poor health. For 69 years and younger, having work change, loss of a close person, and a history of dyslipidemia were associated with the exacerbated oral symptoms. For each of the age categories, there was no statistical evidence that exacerbated oral symptoms were related to experiences of tsunami, hearing nuclear explosions, and job loss; histories of mental illness and diabetes mellitus; and current smoking and exercise habits.

## 4. Discussion

This cross-sectional study of residents of the evacuation zone in Fukushima Prefecture after the Great East Japan Earthquake found an association between disaster-related psychosocial factors and oral symptoms. In particular, the factors associated with exacerbated oral symptoms were having PTSD symptoms; experience of evacuation, hearing nuclear explosions, work changes, loss of a close person; history of dyslipidemia; and subjective current poor health condition. The associations of PTSD symptoms and current poor health conditions with the exacerbated oral symptoms were particularly pronounced, even when stratified by sex and age category. Even 10 to 19 months after the earthquake in this study, the effects of the disaster were still seen in many cases, suggesting that these factors may have psychologically influenced the worsening of oral symptoms.

In this study, the participants with PTSD symptoms had a higher prevalent and exacerbated oral symptoms than those without PTSD symptoms, and the association between PTSD symptoms and exacerbated oral symptoms was particularly strong. As no studies have reported the associations between psychological factors and oral symptoms after a large-scale disaster, the results of this study were difficult to compare with those of previous studies. However, a well-known fact is that stress influences the worsening of oral symptoms [14,22,23]. Chronic stress has been reported to cause high glucocorticoid levels in the blood, suppressed immunity [24], and a lower threshold for chronic pain perception [25]. In patients with stress reactions, periodontal tissue pain has also been reported to be caused by the application of excessive force to the teeth, such as clenching and grinding [26]. It is also well-known that psychological trauma and long-term stress can produce symptoms of temporomandibular joint dysfunction (TMD) such as temporomandibular joint (TMJ) noise, pain, and restricted opening. Ferreira et al. reported a bidirectional relationship between PTSD and painful TMD in their review [27]. Although TMJ symptoms were not investigated in this study, the fact that PTSD was associated with oral symptoms suggests that oral pain may have been affected by the presence of TMD. While approximately two-thirds of patients with PTSD recover over time, others may be more severely affected, and their post-traumatic state may persist, affecting their cognitive and behavioral functions for years or longer [28,29]. Therefore, the association between psychological factors and the exacerbated oral symptoms may be stronger than usual owing to the continuation of chronic stress after a large-scale disaster.

Social factors can also worsen the oral environment [14,22,23]. Factors contributing to psychosocial stress related to the disaster in this study included evacuation, unemployment, and work changes. Greater psychological distress was observed in those who lived in temporary housing for long periods of time due to evacuation [30]. For example, the use of shelters could also cause psychological stress due to changes in available space and noise from neighbors [30,31]. The fact that evacuation was associated with exacerbated oral symptoms in participants suggests that psychological stress caused by evacuation was likely to worsen oral symptoms. Unemployed people have been reported to be more likely to be affected by psychological factors [32], but after adjustment by Model 2, no relationship with job loss was found in this study. It has been reported that after the earthquake, younger people had more negative attitudes toward work, and those whose work was affected in some way by the earthquake had more negative attitudes toward work [33]. In present study, workers under 70 years of age may have had more factors associated with oral symptoms, as changes in the work environment, personal relationships, and work content at the new evacuation site caused greater stress. The results of this study suggest that social factors such as life changes after the disaster, were potentially associated with psychological stress, and such stress might be associated with exacerbated oral symptoms. However, it is important to note that psychological stress may be related to other lifestyle factors such as sleep, social participation, and diet, although smoking and exercise habits were not associated with worsened oral symptoms after the multivariate adjustment in this study. However, it is important to note that psychological stress may also be related to other lifestyle factors such as sleep, social participation, and diet.

Regarding physical factors, the participants with a history of dyslipidemia in this study were more likely to have prevalent and exacerbated oral symptoms than the participants without a history of dyslipidemia, and the association with exacerbated oral symptoms was particularly strong. Dyslipidemia causes abnormalities in immune system cells and wound healing, resulting in increased susceptibility to periodontitis and other infections [34]. In residents of the evacuation zone in Fukushima, lifestyle-related diseases such as obesity and dyslipidemia increased after the earthquake owing to dietary changes and chronic stress [35], which may have aggravated periodontal disease. Dutta-Roy (1994) also reported that dyslipidemia may be associated with the development of many diabetic complications rather than hyperglycemia [36]. In fact, the associations between history of diabetes mellitus and the prevalence and exacerbation of oral symptoms disappeared after adjustment for Model 2 in the present study, which supports the results of previous studies. On the other hand, considering that this study was a cross-sectional study, oral symptoms may have affected the development of dyslipidemia. Recently, dyslipidemia and periodontal disease have been reported to be interrelated [37]. Periodontal disease is associated with increases in the levels of lipopolysaccharides (LPS) and cytokines such as tumor necrosis factor alpha and interleukin 1 [37], and these cytokines may have a negative effect on lipid metabolism [38]. Moreover, periodontal infection (*Porphyromonas gingivalis*) has been associated with abnormal lipid metabolism, which is associated with the progression of atherosclerosis [39]. In addition, it has been reported that periodontitis induces dyslipidemia [40]. Therefore, the relationship between dyslipidemia and oral symptoms must be clarified by prospective studies in the future.

Symptoms such as continuous pain, swelling, and discomfort in the oral cavity affect not only eating but also normal life. The more severe the caries and periodontal diseases, the stronger the subjective symptoms. Furthermore, the weaker the immune system, the lower the resistance to infection and the worse the oral symptoms [41]. Therefore, it is important to maintain oral health in disaster-affected areas where the oral environment is likely to deteriorate. OHRQOL is an index for evaluating oral health based on three factors: “pain and discomfort,” “physical functioning,” and “psychosocial functioning” [42]. There have been many reports on the association between a history of toothache and periodontal disease and OHRQOL [43], as well as the association between subjective general health status and OHRQOL [9,10,44]. In addition, tooth movement and the remaining teeth, which are characteristics of periodontal disease, have been associated with lower OHRQOL, and OHRQOL has been reported to be poor in middle-aged and elderly people in the areas affected by the earthquake [9]. According to results of the 2016 Dental Disease Survey conducted by the Ministry of Health, Labor, and Welfare with 6278 people in Japan, the subjective symptoms of toothache were 15%–20% in those aged 64 years or younger and less than 10% in those aged 65 years or older, while the subjective symptoms of gingivitis were around 15% in those aged 64 years or younger and around 10% in those aged 65 years or older. However, actual oral conditions and subjective symptoms, such as tooth decay and periodontitis, cannot be compared because they progress asymptomatically and there are no studies showing worsening during normal times. In this study, we stratified by age category and found that the middle-aged groups (age 50–69 years) had more factors associated with worsening oral symptoms than other age categories. This is most likely due to the fact that many middle-aged people have many remaining teeth, have periodontal disease, and are more vulnerable to social factors such as work and family. In this study, the proportions of all the participants with oral symptoms or exacerbation were 10.6% in those aged 49 years or younger, 13.1% in those aged 50–69 years, and 11.7% in those aged 70 years or older, which is not consistent with previous reports that people older than 70 years old reported the fewest oral symptoms.

The strengths of this study are the following: (1) to the best of our knowledge, this is the first large-scale study to examine oral symptoms after an environmental disaster epidemiologically and in detail in relation to psychosocial factors, and (2) the recruitment and survey were systematically conducted after the disaster. With regard to the deterioration of the oral environment after a large-scale disaster, it is important for dentists to understand that there is a possibility that the cause is not only oral but also related to the health of the whole body. Furthermore, as a preventive measure against systemic diseases, maintaining the oral environment could prevent the worsening of systemic diseases, such as dyslipidemia.

There are several limitations in this study. First, the overall response rate was low (40.7%), and there were proxy responses (12.6%), which resulted in a relatively small number of respondents for analysis. Moreover, there was a possibility that some of the non-evacuees might have actually been evacuated. It should be noted that the study may have underestimated the association between psychological factors, including experience of evacuation and oral symptoms, as non-respondents were reported to be more likely than respondents to be employed (*p* = 0.02), socially isolated (*p* = 0.047), and to have a high psychological stress response (*p* = 0.03) [45]. Furthermore, the participants in this study were residents of the evacuation zone and did not represent all residents of Fukushima Prefecture. Second, because we did not conduct actual oral examinations and evaluated the data subjectively, the results might differ from the actual oral condition, such as the presence or absence of dental treatment; the number of teeth; and the presence, damage, or loss of dentures and, therefore, could not be compared with actual oral changes or denture restoration. However, since 70% of the participants who had dentures had them restored by 9 months after the Great East Japan Earthquake, and there was no difference between the people of unrestored and restored dentures [9], and since subjective symptoms of caries and periodontal disease worsen as the severity of the disease increases and the immune system is weakened, we believe that similar results could be expected from a subjective assessment. Third, since this was a cross-sectional study, it is not possible to show causality. In the future, it is necessary to confirm the causal relationship by conducting a longitudinal study after further examining the conditions and survey methods.

## 5. Conclusions

In this cross-sectional study of Fukushima residents after the Great East Japan Earthquake, it was found that psychological factors such as PTSD symptoms; social factors such as evacuation, work change, and loss of a close person; and physical factors such as current poor health condition and history of dyslipidemia were associated with exacerbated oral symptoms. Since worsening oral function is a factor in the future development of cardiovascular disease and nursing care requirement, it is necessary to take a preventive approach from each of the physical, psychological, and social aspects in order to prevent disease and need for nursing care in the post-disaster population.

## Figures and Tables

**Table 1:** Prevalence of oral symptoms and basic background information of 64,186 participants.

Variable	Sex		Age Category		
	Men	Women	≤49 Years	50–69 Years	≥70 Years
*n*	27,953 (43.55)	36,233 (56.45)	22,657 (35.3)	26,139 (40.7)	15,390 (24.0)
Age (years, mean ± SD)	56.4 ± 17.5	54.3 ± 17.9	34.8 ± 9.0	60.1 ± 5.4	76.9 ± 5.2
49 years old or younger (*n*, %)	8969 (32.1)	13,688 (37.8)			
50 to 69 years (*n*, %)	11,916 (42.6)	14,223 (39.3)			
70 years old or older (*n*, %)	7068 (25.3)	8322 (23.0)			
Disaster-related factors					
PTSD symptoms (*n*, %)	4848 (18.1)	8217 (24.2)	4001 (17.8)	5379 (21.4)	3685 (28.3)
Psychological distress (*n*, %)	3132 (11.9)	5585 (16.7)	3124 (14.0)	3656 (14.8)	1937 (15.3)
Experience of evacuation (*n*, %)	13,170 (47.1)	16,835 (46.5)	11,147 (49.2)	12,239 (46.8)	6619 (43.0)
Experience of tsunami (*n*, %)	6718 (24.0)	6314 (17.4)	4160 (18.4)	5164 (19.8)	3708 (24.1)
Experience of nuclear accident (explosion heard) (*n*, %)	15,465 (55.3)	18,301 (50.5)	10,458 (46.2)	14,127 (54.1)	9181 (59.7)
House damage (major) (%)	4157 (15.8)	5339 (15.9)	2911 (14.0)	4107 (16.7)	2478 (17.3)
Job loss (*n*, %)	5142 (18.4)	8285 (22.9)	5289 (23.3)	6452 (24.7)	1686 (11.0)
Work change (*n*, %)	15,095 (56.7)	18,270 (55.0)	13,249 (59.5)	14,491 (57.8)	5625 (44.8)
Loss of a close person (*n*, %)	5153 (19.0)	7317 (20.8)	3827 (17.1)	5363 (20.9)	3280 (22.9)
Medical history					
Mental illness (*n*, %)	1137 (4.2)	1942 (5.6)	1144 (5.2)	1225 (4.8)	710 (5.0)
Hypertension (*n*, %)	12,625 (45.8)	13,385 (37.6)	2464 (10.9)	12,440 (48.4)	11,106 (74.6)
Diabetes mellitus (*n*, %)	6327 (23.3)	5755 (16.5)	1047 (4.7)	5675 (22.3)	5360 (38.1)
Dyslipidemia (*n*, %)	10,219 (37.6)	12,106 (34.5)	3241 (14.4)	11,572 (45.3)	7512 (52.8)
Current poor health condition (*n*, %)	4798 (17.5)	6817 (19.3)	2836 (12.6)	5109 (19.9)	3670 (24.9)
Lifestyle factors					
Current smoking habit (*n*, %)	9247 (33.5)	3912 (11.3)	6836 (30.3)	5138 (20.2)	1185 (8.3)
Current drinking habit (*n*, %)	18,539 (66.9)	10,437 (30.0)	10,632 (47.1)	13,088 (50.8)	5256 (36.3)
Current exercise habit (once a week or less) (*n*, %)	16,850 (61.7)	23,620 (67.0)	18,374 (81.5)	16,410 (63.9)	5686 (39.7)
Oral symptoms					
Absent (*n*, %)	24,802 (88.7)	31,769 (87.7)	20,273 (89.5)	22,713 (86.9)	13,585 (88.3)
Prevalent (*n*, %)	2775 (9.9)	3827 (10.6)	1985 (8.8)	2986 (11.4)	1631 (10.6)
Exacerbated (*n*, %)	376 (1.4)	637 (1.8)	399 (1.8)	440 (1.7)	174 (1.1)

Data are presented as means with the standard deviations or numbers with the proportions. SD: standard deviation, PTSD: post-traumatic stress disorder.

**Table 2:** Adjusted odds ratios and 95% CI for prevalent and for exacerbated oral symptoms.

Variables	Model 1 ^1^ ORs (95% CI)	Model 2 ^2^ ORs (95% CI) ^1^
Prevalent		
Disaster-related factors		
PTSD symptoms (Ref. no)	**2.59** (2.45–2.74)	**1.80** (1.57–2.07)
Work change (Ref. no)	**1.47** (1.39–1.55)	**1.18** (1.04–1.35)
Experience of nuclear accident (explosion heard) (Ref. no)	**1.46** (1.38–1.53)	**1.18** (1.05–1.33)
Loss of a close person (Ref. no)	**1.44** (1.36–1.53)	**1.15** (1.01–1.32)
Experience of evacuation (Ref. no)	**1.37** (1.30–1.44)	1.06 (0.95–1.20)
Job loss (Ref. no)	**1.36** (1.28–1.45)	1.01 (0.87–1.17)
Experience of tsunami (Ref. no)	**1.33** (1.26–1.42)	0.93 (0.79–1.08)
House damage (Ref. minor or none)	**1.27** (1.19–1.36)	1.02 (0.86–1.21)
Medical history		
Mental illness (Ref. no)	**2.43** (2.22–2.67)	**1.33** (1.07–1.65)
Dyslipidemia (Ref. no)	**1.41** (1.33–1.49)	**1.32** (1.15–1.50)
Diabetes mellitus (Ref. no)	**1.28** (1.20–1.37)	0.85 (0.72–1.01)
Hypertension (Ref. no)	**1.25** (1.18–1.33)	**1.20** (1.05–1.38)
Current poor health condition (Ref. good)	**3.10** (2.93–3.28)	**2.12** (1.85–2.44)
Lifestyle factors		
Current exercise habit (once a week or less) (Ref. twice a week or more)	**1.14** (1.08–1.21)	1.10 (0.97–1.25)
Current smoking habit (Ref. no)	**1.08** (1.01–1.15)	1.05 (0.92–1.21)
Women (Ref. men)	**1.09** (1.04–1.15)	1.03 (0.91–1.16)
Age categories (Ref. ≥70 years)		
≤49 years	**0.77** (0.72–0.81)	0.98 (0.80–1.20)
50–69 years	**1.24** (1.17–1.30)	1.17 (0.98–1.40)
**Exacerbated**	**Model 1 ^1^ ORs (95% CI)**	**Model 2 ^2^ ORs (95% CI)**
Disaster-related factors		
PTSD symptoms (Ref. no)	**4.07** (3.58–4.64)	**2.24** (1.64–3.06)
Work change (Ref. no)	**2.32** (2.01–2.69)	**1.88** (1.34–2.65)
Loss of a close person (Ref. no)	**1.92** (1.67–2.20)	1.25 (0.92–1.71)
Job loss (Ref. no)	**1.84** (1.61–2.11)	1.11 (0.81–1.51)
Experience of evacuation (Ref. no)	**1.84** (1.62–2.09)	1.20 (0.91–1.58)
Experience of nuclear accident (explosion heard) (Ref. no)	**1.76** (1.55–2.01)	1.11 (0.84–1.47)
House damage (Ref. minor or none)	**1.58** (1.35–1.85)	1.19 (0.82–1.72)
Experience of tsunami (Ref. no)	**1.31** (1.13–1.52)	1.14 (0.81–1.60)
Medical history		
Mental illness (Ref. no)	**2.33** (1.87–2.89)	0.98 (0.60–1.62)
Dyslipidemia (Ref. no)	**1.66** (1.45–1.91)	**1.74** (1.27–2.39)
Hypertension (Ref. no)	**1.32** (1.14–1.53)	0.99 (0.70–1.42)
Diabetes mellitus (Ref. no)	**1.21** (1.02–1.44)	0.76 (0.48–1.20)
Current poor health condition (Ref. good)	**4.27** (3.75–4.87)	**2.73** (2.00–3.75)
Lifestyle factors		
Current smoking habit (Ref. no)	**1.28** (1.09–1.49)	1.09 (0.80–1.48)
Current exercise habit (once a week or less) (Ref. twice a week or more)	**1.20** (1.04–1.38)	1.09 (0.79–1.52)
Women (Ref. men)	**1.31** (1.15–1.49)	1.10 (0.83–1.47)
Age categories (Ref. ≥70 years)		
≤49 years	**1.14** (1.01–1.30)	**3.83** (1.90–7.73)
50–69 years	**1.16** (1.02–1.31)	**2.48** (1.26–4.88)

^1^ Model 1: adjusted for sex and age category. ^2^ Model 2: adjusted for sex and age category + all variables that were significant in Model 1. Bold considers statistically significant. CI: confidence interval, OR: odds ratio, Ref: reference, PTSD: post-traumatic stress disorder.

**Table 3:** Multiple adjusted odds ratios and 95% CI for prevalent and for exacerbated oral symptoms by sex and age category.

Variables	Sex		Age Category		
Prevalent	Men ^1^	Women ^1^	≤49 Years ^2^	50–69 Years ^2^	≥70 Years ^2^
*n*	21,957	26,060	19,014	20,420	8583
Women (Ref. men)			1.05 (0.94–1.18)	1.01 (0.92–1.12)	1.02 (0.88–1.18)
Age category (Ref. ≥70 years)					
≤49 years	0.99 (0.85–1.15)	0.98 (0.84–1.13)			
50–69 years	**1.15** (1.02–1.31)	**1.17** (1.03–1.32)			
Disaster factors					
PTSD symptoms (Ref. no)	**1.81** (1.62–2.02)	**1.72** (1.57–1.90)	**1.74** (1.53–1.99)	**1.77** (1.59–1.96)	**1.82** (1.55–2.14)
Experience of evacuation (Ref. no)	**1.16** (1.05–1.28)	1.08 (0.99–1.18)	1.02 (0.91–1.13)	**1.21** (1.10–1.33)	1.08 (0.92–1.26)
Experience of tsunami (Ref. no)	1.09 (0.98–1.22)	1.04 (0.94–1.17)	1.02 (0.89–1.17)	1.01 (0.90–1.13)	**1.28** (1.08–1.51)
Experience of nuclear accident (explosion heard) (Ref. no)	**1.14** (1.04–1.26)	**1.11** (1.01–1.21)	1.08 (0.97–1.21)	**1.12** (1.02–1.23)	**1.24** (1.06–1.47)
House damage (Ref. minor or none)	0.95 (0.83–1.07)	0.98 (0.87–1.10)	0.93 (0.80–1.09)	1.07 (0.95–1.21)	**0.77** (0.63–0.94)
Job loss (Ref. no)	**1.15** (1.02–1.30)	0.96 (0.86–1.07)	1.07 (0.94–1.23)	1.04 (0.93–1.16)	0.89 (0.71–1.12)
Work change (Ref. no)	**1.14** (1.03–1.27)	**1.18** (1.07–1.30)	**1.21** (1.07–1.37)	**1.16** (1.04–1.29)	1.10 (0.93–1.30)
Loss of a close person (Ref. no)	1.09 (0.97–1.22)	**1.17** (1.06–1.29)	1.11 (0.97–1.26)	**1.14** (1.02–1.27)	1.17 (0.99–1.38)
Medical history					
Mental illness (Ref. no)	**1.40** (1.17–1.69)	**1.37** (1.18–1.59)	1.10 (0.90–1.34)	**1.59** (1.34–1.90)	**1.56** (1.19–2.04)
Dyslipidemia (Ref. no)	**1.26** (1.13–1.40)	**1.14** (1.03–1.26)	**1.22** (1.05–1.43)	**1.19** (1.08–1.31)	**1.18** (1.00–1.39)
Hypertension (Ref. no)	1.01 (0.91–1.13)	1.05 (0.94–1.17)	1.10 (0.93–1.31)	1.03 (0.93–1.13)	0.94 (0.79–1.11)
Diabetes mellitus (Ref. no)	1.05 (0.93–1.19)	1.06 (0.93–1.20)	1.24 (0.98–1.58)	1.04 (0.93–1.17)	1.04 (0.87–1.23)
Current poor health condition (Ref. good)	**2.32** (2.08–2.59)	**2.19** (1.99–2.42)	**2.19** (1.91–2.52)	**2.15** (1.94–2.39)	**2.49** (2.13–2.91)
Lifestyle factors					
Current smoking habit (Ref. no)	1.01 (0.91–1.12)	1.00 (0.88–1.14)	1.10 (0.98–1.23)	0.97 (0.86–1.09)	0.79 (0.59–1.06)
Current exercise habit (once a week or less) (Ref. twice a week or more)	**1.13** (1.02–1.25)	1.04 (0.94–1.14)	1.04 (0.90–1.19)	1.09 (0.99–1.19)	1.10 (0.95–1.28)
**Exacerbated**	**Men ^1^**	**Women ^1^**	**≤49 years ^2^**	**50–69 years ^2^**	**≥70 years ^2^**
*n*	20,087	23,825	17,712	18,425	7775
Women (Ref. men)			1.16 (0.90–1.49)	**1.41** (1.10–1.80)	0.89 (0.57–1.38)
Age category (Ref. ≥70 years)					
≤49 years	**1.78** (1.18–2.67)	**2.04** (1.38–2.99)			
50–69 years	1.22 (0.85–1.76)	**1.82** (1.29–2.89)			
Disaster factors					
PTSD symptoms (Ref. no)	**3.10** (2.39–4.03)	**2.33** (1.88–2.89)	**2.99** (2.32–3.86)	**2.24** (1.75–2.87)	**2.79** (1.75–4.46)
Experience of evacuation (Ref. no)	1.24 (0.97–1.60)	1.20 (0.98–1.47)	0.94 (0.75–1.19)	**1.38** (1.08–1.76)	**2.08** (1.29–3.34)
Experience of tsunami (Ref. no)	0.77 (0.58–1.02)	1.00 (0.78–1.27)	0.92 (0.69–1.22)	0.91 (0.68–1.20)	0.78 (0.47–1.31)
Experience of nuclear accident (explosion heard) (Ref. no)	**1.46** (1.12–1.91)	1.06 (0.87–1.30)	1.14 (0.90–1.44)	1.21 (0.95–1.54)	1.41 (0.84–2.36)
House damage (Ref. minor or none)	1.05 (0.78–1.42)	1.22 (0.96–1.56)	1.10 (0.81–1.48)	**1.32** (1.00–1.73)	0.84 (0.49–1.45)
Job loss (Ref. no)	0.95 (0.71–1.25)	1.15 (0.91–1.44)	1.13 (0.87–1.46)	0.91 (0.70–1.19)	1.26 (0.70–2.25)
Work change (Ref. no)	**1.95** (1.43–2.64)	**1.52** (1.19–1.94)	**1.84** (1.36–2.48)	**1.79** (1.34–2.38)	1.11 (0.68–1.84)
Loss of a close person (Ref. no)	1.31 (0.998–1.72)	**1.50** (1.21–1.86)	**1.31** (1.00–1.70)	**1.53** (1.20–1.95)	1.53 (0.96–2.44)
Medical history					
Mental illness (Ref. no)	0.78 (0.49–1.26)	1.14 (0.82–1.58)	0.73 (0.48–1.11)	1.31 (0.88–1.96)	1.03 (0.46–2.33)
Dyslipidemia (Ref. no)	**1.59** (1.23–2.07)	**1.40** (1.11–1.78)	**1.68** (1.25–2.26)	**1.32** (1.04–1.67)	1.61 (0.98–2.65)
Hypertension (Ref. no)	0.98 (0.74–1.30)	1.03 (0.80–1.32)	**1.46** (1.05–2.03)	0.79 (0.62–1.01)	1.16 (0.67–2.01)
Diabetes mellitus (Ref. no)	0.80 (0.58–1.11)	0.90 (0.66–1.23)	0.82 (0.51–1.33)	0.84 (0.62–1.14)	0.87 (0.53–1.43)
Current poor health condition (Ref. good)	**2.90** (2.22–3.77)	**2.40** (1.93–3.00)	**2.58** (1.98–3.36)	**2.65** (2.06–3.42)	**2.14** (1.36–3.38)
Lifestyle factors					
Current smoking habit (Ref. no)	**1.30** (1.02–1.67)	1.01 (0.77–1.33)	1.15 (0.91–1.47)	1.14 (0.85–1.52)	1.03 (0.46–2.29)
Current exercise habit (once a week or less) (Ref. twice a week or more)	1.00 (0.77–1.31)	1.17 (0.93–1.46)	1.28 (0.93–1.77)	0.92 (0.73–1.17)	1.40 (0.91–2.15)

^1^ Adjusted for age category + everything else. ^2^ Adjusted for sex + everything else. Bold considers statistically significant. CI: confidence interval, OR: odds ratio, Ref: reference, PTSD: post-traumatic stress disorder.

## Data Availability

The datasets analyzed during the present study are not publicly available because the data from the Fukushima Health Management Survey belongs to the government of Fukushima Prefecture and can only be used within the organization.

## Supplementary Materials

The following are available online at https://www.mdpi.com/article/10.3390/ijerph18116054/s1, Supplementary S1. Fukushima Health Management Survey Mental Health and Lifestyle Survey (for adults) 2011.
