# Supplementary material for: Association between Psychosocial Factors and Oral Symptoms among Residents in Fukushima after the Great East Japan Earthquake: A Cross-Sectional Study from the Fukushima Health Management Survey
Source: Int J Environ Res Public Health. 2021 Jun 4;18(11):6054. doi: 10.3390/ijerph18116054 (PMC8200098; doi:10.3390/ijerph18116054)
Supplement: Supplementary file 1 [file ijerph-18-06054-s001.zip › ijerph-1150984-supplementary.pdf]

2011

Fukushima Health Management Survey
Mental Health and Lifestyle Survey
(For Adults)

The nuclear accidents accompanying the recent earthquake and tsunami have caused a great deal of anxiety and stress, with many people forced to lead dramatically altered lifestyles in evacuation shelters and temporary housing.

Fukushima Health Management Survey aims to assess the physical and mental condition of residents from the evacuation zone in order to facilitate appropriate health care in the future.

This questionnaire booklet is being distributed to those born on or before 1 April 1995.

Any personal information that you provide in this questionnaire will be used by Fukushima Prefecture to administer health care and may also be shared with your local municipal government where necessary, with findings reported only as part of an aggregate analysis. Personal information will only be disclosed in a manner that protects your identity. Please complete the questionnaire on your own. If you are under 20 years old you must obtain the consent of a parent or legal guardian so please have your parent/guardian sign the space provided on the following page before filling out this form. If you are unable to complete the questionnaire on your own due to old age or disability, please have it filled out by a family member or housemate.

Please return your completed questionnaire form within approximately 2 weeks of receiving it.

Any queries should be directed to the contacts listed on the back of this booklet.

Fukushima Medical University
Fukushima Prefecture

Please fill out all of the items below, and place a tick (✔) in the appropriate box.

Date (YY/MM/DD): 2012/ /	Respondent: 1☐ Yourself<br>2☐ Proxy (Relationship:______________)
Name:____	Sex: 1☐ Male 2☐ Female
Date of birth (YY/MM/DD): 1☐Meiji ･2☐ Taisho 3☐ Showa ･4☐ Heisei / /	
Signature of Legal Guardian (required for underage respondents):<br><br>Signature:____	
Registered address on March 11, 2011 according to Certificate of Residence/Alien Registration:<br>☐ Same as questionnaire mailing address (address not required)<br>〒______ -______ ______________<br>_______________ Metropolis/Circuit Prefecture ___________ Ward/City _____________ Ward/Town Village ________________________________<br>Apartment Name & No.____	
Current address: 1☐ Same as questionnaire mailing address (address not required)<br>2☐ Same as Certificate of Residence/Alien Registration address (address not required)<br>〒______ -______<br>_______________ Metropolis/Circuit Prefecture ___________ Ward/City _____________ Ward/Town Village ________________________________<br>Apartment Name & No.____	
Intended new address (fill out this section if you know your new address):<br>〒______ -______<br>_______________ Metropolis/Circuit Prefecture ___________ Ward/City _____________ Ward/Town Village ________________________________<br>Apartment Name & No.____<br>Scheduled moving date (YY/MM/DD): / /	
Contact details (these details are required so that a survey officer can contact you directly to confirm any omissions in the questionnaire).<br>Tel:(______)________―________ (Care of:_______________________)<br>Mobile: ______―_______-________	
Type of work: 1☐ Full time 2☐ Part-time 3☐ Unemployed (includes students & homemakers)	

Q1. Describe your current state of health (tick (✔) the relevant box below).

Very good	Good	Normal	Poor	Very poor
1☐	2☐	3☐	4☐	5☐

Q2. Have you ever been diagnosed with the following conditions?

1) Hypertension or high blood pressure

1☐ No 2☐ Yes ➡ Are you currently visiting a hospital for treatment?

2) Diabetes or high blood sugar

1☐ No 2☐ Yes ➡ Are you currently visiting a hospital for treatment?

3) Hyperlipidemia or high cholesterol/ triglycerides

1☐ No 2☐ Yes ➡ Are you currently visiting a hospital for treatment?

4) Cancer (including leukemia or lymphoma)

1☐ No 2☐ Yes ➡ What type of cancer? (Please list all types)

______________ ; _______________; ______________

5) Stroke

1☐ No 2☐ Yes ➡ What type of stroke? (You can tick more than 1 box)

1☐ Cerebral infarction (embolism; thrombus)

Caused by a blocked blood vessel in the brain

2☐ Cerebral hemorrhage 3☐ Subarachnoid hemorrhage

4☐ Other(_____________) 5☐ Not sure

6) Heart disease

1☐ No 2☐ Yes ➡ What type of heart disease? (You can tick more than 1 box)

1☐ Myocardial infarction

Caused by a blocked blood vessel in the heart

2☐ Angina pectoris

3☐ Other (_____________) 4☐ Not sure

7) Chronic hepatitis

1☐ No 2☐ Yes ➡ What type of chronic hepatitis?

1☐ Hepatitis B 2☐ Hepatitis C

3☐ Other (_____________)

8) Pneumonia (in the past 10 years)

1☐ No   2☐ Yes

9) Bone fracture after the age of 50

1☐ No   2☐ Yes

10) Thyroid disease

1☐ No   2☐ Yes → What type of thyroid disease?

1☐ Hyperthyroidism (Basedow's disease)

2☐ Hypothyroidism

3☐ Other (____________)

11) Mental illness

1☐ No   2☐ Yes

Q3. Have you ever undergone the following medical tests? Tick (✔) the relevant box below.

1. CT scan (a test using a large medical scanning device with a hollow center that is much quieter than an MRI scanner)

1☐ No

2☐ Yes

3☐ Not sure

2. Fluoroscopy (a test using white barium contrast agent)

1☐ No

2☐ Yes

3☐ Not sure

3. Angiography (e.g. cardiac catheter test), nuclear medicine test (scintigraphy), or PET scan

1☐ No

2☐ Yes → 1☐ Angiography   2☐ Nuclear medicine test   3☐ PET scan

3☐ Not sure

Q4. Have you ever undergone radiation therapy as part of your medical treatment? Tick (✔) the relevant box below.

$_1$☐ No $_2$☐ Yes →

$_3$☐ Not sure

Indicate each age if you underwent radiotherapy more than once

What illness were you treated for?	How old were you?
______________________	☐ years old
______________________	☐ years old
______________________	☐ years old

Q5. Have you felt unwell <u>in the past several days</u> due to illness or injury etc.?

$_1$☐ Yes $_2$☐ No

↓

What kind of symptoms have you had? Please circle **all** applicable symptoms in the box below, and place a double circle (◎) around any symptoms that have worsened since the disaster.

a. Sore wrists or ankles	b. Easily irritated	c. Headache
d. Dizziness	e. Palpitations	f. Shortness of breath
g. Coughing and/or phlegm	h. Wheezing	i. Diarrhea
j. Constipation	k. Loss of appetite	l. Abdominal/ stomach pain
m. Hemorrhoid pain/ bleeding	n. Toothache	o. Swollen/ bleeding gums
p. Difficulty chewing	q. Itching (rash, athlete's foot, etc.)	r. Lower back pain

s. Inco **Q1. Describe your current state of health (tick (✔) the relevant box below).**

Very good	Good	Normal	Poor	Very poor

Q6. Describe your activities of daily living.

1) Please indicate whether you can perform the following tasks on your own.

Routine daily activity	Can do by myself	Can't do by myself
1. Eat meals without assistance (does not include meal preparation)	$_1$☐	$_2$☐
2. Get dressed without assistance	$_1$☐	$_2$☐
3. Go to the toilet without assistance	$_1$☐	$_2$☐
4. Go shopping for daily necessities	$_1$☐	$_2$☐

2) Do you participate in recreation (karaoke or gateball, etc.) and community activities (festivals, etc.)? Tick (✔) the relevant box below.

$_1$☐ Never or rarely $_2$☐ Sometimes $_3$☐ Often

Q7. Describe your sleeping habits.

1) Are you satisfied with the quality of your sleep over the past month (regardless of sleep duration)? Tick (✔) the relevant box below.

$_1$☐ Satisfied $_2$☐ Slightly dissatisfied

$_3$☐ Quite dissatisfied $_4$☐ Very dissatisfied or haven't slept at all

2) Have you experienced any of the following at least 3 times a week?

		Yes	No
1	Takes time to fall asleep after going to bed at night	$_1$☐	$_2$☐
2	Wake up in the middle of the night	$_1$☐	$_2$☐
3	Wake up earlier than planned and can't go back to sleep	$_1$☐	$_2$☐
4	Not enough sleep	$_1$☐	$_2$☐
5	Feel depressed during the day	$_1$☐	$_2$☐
6	Reduced mental and physical activity during the day	$_1$☐	$_2$☐
7	Feel tired during the day	$_1$☐	$_2$☐

Q8. Smoking

1) Do you smoke cigarettes (excluding cigars and pipes)? Tick (✔) the relevant box below.

$_1$☐ Never smoked $_2$☐ Quit $_3$☐ Current smoker

How long since you quit? ☐ year(s) ☐ month(s)

Proceed to 2)

On average, how many cigarettes do you smoke each day?

* If you quit, how many did you used to smoke?

About ☐ cigarettes/day

How many years have you smoked in total?

About ☐ years

2) Over the past 10 years, have you been exposed to passive cigarette smoke in your home or workplace? Tick (✔) the relevant box below.

$_1$☐ Daily $_2$☐ About 4-5 times/week $_3$☐ Sometimes $_4$☐ Rarely

3) Did you smoke before the disaster on March 11, 2011? Tick (✔) the relevant box below.

$_1$☐ No $_2$☐ Yes

Q9. Alcohol consumption

1) Do you drink alcohol? Tick (✔) the relevant box below.

1☐ Don't drink or only rarely (less than once/month) → Proceed to 2)

2☐ Quit → How long since you quit? ☐ year(s) ☐ month(s) → Proceed to 2)

3☐ Drink (at least once a month) → Proceed to 2)

Describe the frequency and average amount of your alcohol intake over the past year.

Type		Don't drink	Frequency (tick (✔) relevant box)					Amount per time
			Less than once/week	1-2 times/ week	3-4 times/ week	5-6 times/ week	Daily	
Beer	Large bottle	0☐	1☐	2☐	3☐	4☐	5☐	____bottles
	Medium bottle/can	0☐	1☐	2☐	3☐	4☐	5☐	____bottles
	Small bottle/can	0☐	1☐	2☐	3☐	4☐	5☐	____bottles
Sake		0☐	1☐	2☐	3☐	4☐	5☐	____flasks
Shochu		0☐	1☐	2☐	3☐	4☐	5☐	____cups
Wine		0☐	1☐	2☐	3☐	4☐	5☐	____glasses
Spirits*	Single	0☐	1☐	2☐	3☐	4☐	5☐	____glasses
	Double	0☐	1☐	2☐	3☐	4☐	5☐	____glasses

* Whiskey, brandy, and other spirits

2) Did you drink alcohol before the disaster on March 11, 2011? Tick (✔) the relevant box below.

1☐ Didn't drink or only rarely (less than once a month)

2☐ Drank (at least once a month)

Q10. Describe your eating habits. Specifically, how often do you eat/drink the following foods/beverages? **Tick (✔) the relevant box below.**

Food/beverage (Frequency)		Never	Less than once/week	1-2 times/ week	3-4 times/ week	5-6 times/ week	Daily
Rice		0☐	1☐	2☐	3☐	4☐	5☐
Bread		0☐	1☐	2☐	3☐	4☐	5☐
Fish (*sashimi*; cooked/boiled/fried fish, etc.)		0☐	1☐	2☐	3☐	4☐	5☐
Meat	Chicken	0☐	1☐	2☐	3☐	4☐	5☐
	Beef, pork,	0☐	1☐	2☐	3☐	4☐	5☐
	ham, sausages	0☐	1☐	2☐	3☐	4☐	5☐
Vege-tables	Green vegetables (spinach, *komatsuna* Japanese mustard spinach, *nira* leek, etc.)	0☐	1☐	2☐	3☐	4☐	5☐
	Red & orange vegetables (tomatoes, carrots, pumpkins, etc.)	0☐	1☐	2☐	3☐	4☐	5☐
	Light-colored vegetables (Chinese cabbage, cabbage, *daikon* radish)	0☐	1☐	2☐	3☐	4☐	5☐
	Vegetable juice	0☐	1☐	2☐	3☐	4☐	5☐
Fruits	Fruits	0☐	1☐	2☐	3☐	4☐	5☐
	Fruit juice	0☐	1☐	2☐	3☐	4☐	5☐
Soy beans	*Natto* fermented soybeans	0☐	1☐	2☐	3☐	4☐	5☐
	Miso soup	0☐	1☐	2☐	3☐	4☐	5☐
	Tofu dishes	0☐	1☐	2☐	3☐	4☐	5☐
	Boiled bean dishes	0☐	1☐	2☐	3☐	4☐	5☐
Milk		0☐	1☐	2☐	3☐	4☐	5☐
Soy milk		0☐	1☐	2☐	3☐	4☐	5☐
Yogurt, lactobacillus drinks		0☐	1☐	2☐	3☐	4☐	5☐

Q11. Do you exercise regularly? Tick (✔) the relevant box below.

1☐ Almost every day 2☐ 2-4 times/week

3☐ Once/week 4☐ Almost never

Q12. How often have you experienced the following in the past 30 days?

Circle (○) the relevant number below.

		Never	Rarely	Some-times	Often	Always
1	Feelings of nervousness	0	1	2	3	4
2	Feelings of hopelessness	0	1	2	3	4
3	Feelings of restlessness and inability to relax	0	1	2	3	4
4	Feelings of inescapable sadness	0	1	2	3	4
5	Feeling that everything requires painstaking effort	0	1	2	3	4
6	Feelings of a lack of self worth	0	1	2	3	4

Q13. Describe your experience of the Great East Japan Earthquake (earthquake, tsunami & nuclear accidents)

<u>About your responses</u>: the following is a list of problems and complaints that may occur after a stressful experience. Please read each item carefully and circle the number of the response that best describes how you have been affected <u>in the past month</u>.

		Not at all	Slightly	Moderately	Quite a lot	Very much
1	Recurring memories, thoughts or images (scenes, etc.) of stressful experiences	1	2	3	4	5
2	Recurrent disturbing dreams of stressful experiences	1	2	3	4	5
3	Behaving or feeling as though a stressful experience is suddenly happening again	1	2	3	4	5
4	Becoming very upset when recalling a stressful experience	1	2	3	4	5
5	Having a physical reaction (e.g. pounding heart, shortness of breath, sweating) when remembering a stressful experience	1	2	3	4	5
6	Trying to avoid thinking or talking about a stressful experience or trying not to have any emotions about the experience	1	2	3	4	5
7	Avoiding certain activities or situations that may remind you of a stressful experience	1	2	3	4	5
8	Unable to recall the important parts of a stressful experience	1	2	3	4	5
9	Losing interest in activities that you previously found enjoyable	1	2	3	4	5

10	Feeling distant or alienated from others	1	2	3	4	5
11	Feeling emotionally numb or unable to feel affection for those close to you	1	2	3	4	5
12	Feeling that you don't have much of a future	1	2	3	4	5
13	Having trouble falling sleep and waking up during the night	1	2	3	4	5
14	Feeling irritated and having angry outbursts	1	2	3	4	5
15	Unable to concentrate	1	2	3	4	5
16	Becoming overly alert, cautious, or careful	1	2	3	4	5
17	Feeling nervous and easily startled	1	2	3	4	5

Q14. Describe how you have been affected by Great East Japan Earthquake.

1) Which of the following disasters did you experience? Tick all applicable boxes.

   1☐ Earthquake 2☐ Tsunami 3☐ Nuclear reactor accident (heard the explosion) 4☐ None

2) What was the result of the government's building damage assessment of your home or residence?

   1☐ No damage 2☐ Partial damage 3☐ Partial collapse

   4☐ Partial but extensive collapse 5☐ Total collapse

3) Did you lose someone close to you in the disaster?

   1☐ Yes 2☐ No

   → Who did you lose? ________________________(____ people)

4) How have your living arrangements changes? Please tick (✔) the relevant box and circle (○) your current residence.

   1☐ Evacuation shelter 2☐ Temporary housing 3☐ Rental housing/ apartment

   4☐ Relative's home 5☐ Own home

   6☐ Other (___________________________________)

5) Has your work situation changed as a result of the natural disaster or nuclear accident?

   1☐ Yes 2☐ No

   How has it changed? Tick (✔) the relevant box below.

   1☐ Started a new job 2☐ Became unemployed 3☐ Changed jobs

   4☐ Income has increased 5☐ Income has decreased

   6☐ Other (___________________________________)

Q15. Describe your current understanding of how radiation exposure has affected your health. **Circle the number of the response that best describes your understanding.**

		Very unlikely			Very likely
1	What do you think is the likelihood of having acute health damage (e.g. dying within 1 month) as a result of your current level of radiation exposure?	1	2	3	4
2	What do you think is the likelihood of damage to your health (e.g. cancer onset) in later life as a result of your current level of radiation exposure?	1	2	3	4
3	What do you think is the likelihood that the health of your future (i.e. as yet unborn) children and grandchildren will be affected as a result of your current level of radiation exposure?	1	2	3	4

* The following questions are intended to obtain information about your health and lifestyle habits, and are not intended to invade your privacy in any way. Please answer to the extent that you feel comfortable.

Q16. Tick (✔) the box below that describes your highest level of education.

1☐ Elementary school・Junior high school 2☐High school

3☐ Vocational college/ Junior college 4☐ University (4 years)・Graduate school

Q17. Have any of your family members (blood relatives) ever had the following diseases? **Place a tick (✔) in the relevant boxes.**

1) Has your biological father ever had the following diseases? (You can tick more than 1 box)

1☐ Stroke 2☐ Heart disease

3☐ Diabetes 4☐ Cancer (Tumor site(s): ; ;)

5☐ Father has never had the above diseases 6☐ Not sure

2) Has your biological mother ever had the following diseases? (You can tick more than 1 box)

1☐ Stroke 2☐ Heart disease

3☐ Diabetes 4☐ Cancer (Tumor site(s): ; ;)

5☐ Mother has never had the above diseases 6☐ Not sure

3) Has your biological brother ever had the following diseases? (You can tick more than 1 box).

Tick (✔) this box if you do not have a biological brother

$_0$☐ Don't have a biological brother

$_1$☐ Stroke $_2$☐ Heart disease

$_3$☐ Diabetes $_4$☐ Cancer (Tumor site(s): ; ;)

$_5$☐ Brother has never had the above diseases $_6$☐ Not sure

4) Has your biological sister ever had the following diseases? (You can tick more than 1 box).

Tick (✔) this box if you do not have a biological sister

$_0$☐ Don't have a biological sister

$_1$☐ Stroke $_2$☐ Heart disease

$_3$☐ Diabetes $_4$☐ Cancer (Tumor site(s): ; ;)

$_5$☐ Sister has never had the above diseases $_6$☐ Not sure

Male respondents: you have now completed the questionnaire. Please return the booklet in the provided return envelope.

Female respondents: please proceed to the next question.
The following questions are intended for women only.

Q18. About menstruation

1) Please describe your current period. Tick (✔) the relevant box below.

$_1$☐ I have periods

$_2$☐ I have reached menopause (incl. surgery-induced menopause)

$_3$☐ I am not menstruating because of pregnancy/childbirth

$_4$☐ I do not have periods due to other reason(s)

$_5$☐ I have never had a period

When did you first get your period? ☐ years old

2) This question is for women who have reached menopause.

① How old were you when your periods ended? ☐ years old

② Why did your period end?

$_1$☐ Natural menopause

$_2$☐ Treatment-induced menopause (oophorectomy; hysterectomy, radiotherapy, chemotherapy, etc.)

$_3$☐ Other ( )

Q19. Have you ever given birth?

1☐ Yes 2☐ No

You have now completed the questionnaire. Please return this questionnaire booklet in the return envelope provided.

Fukushima Health Management Survey

Fukushima Medical University

Queries

For questions on how to request or fill out the questionnaire booklet, please contact:

Radiation Medical Science Center, Fukushima Medical University

Tel: 024-549-5170 (Office hours: 9:00 a.m. - 5:00 p.m.)
